# Supplementary material for: Splice-Junction-Based Mapping of Alternative Isoforms in the Human Proteome
Source: Cell Rep. Author manuscript; Available in PMC 2020 Jan 15. (PMC6961840; doi:10.1016/j.celrep.2019.11.026)
Supplement: 2 [file NIHMS1546469-supplement-2.zip › DF1/PXD006675/CardiacFibroblast/CardiacFibroblast_20_BCAP29_EHSELQVPLGSFYILAFAPGLHNPHPSSPR.pdf]

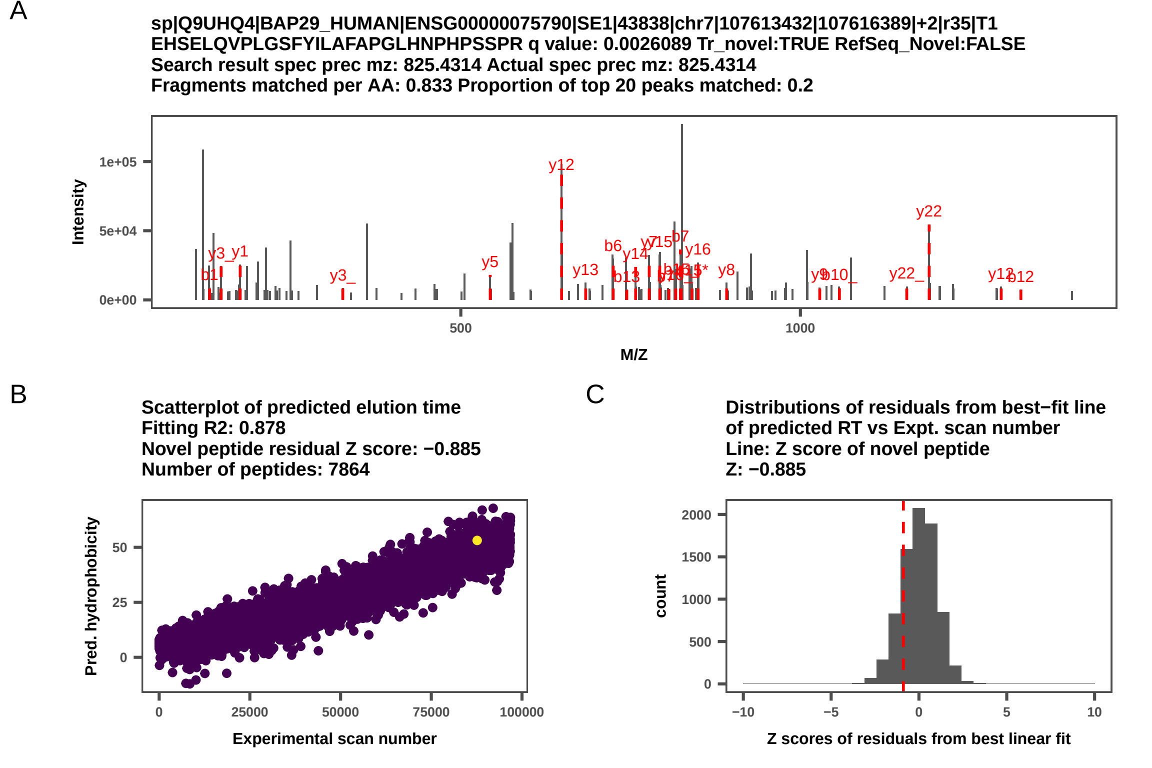
A
sp|Q9UHQ4|BAP29_HUMAN|ENSG00000075790|SE1|43838|chr7|107613432|107616389|+2|r35|T1
EHSELQVPLGSFYILAFAPGLHNPHPSSPR q value: 0.0026089 Tr_novel:TRUE RefSeq_Novel:FALSE
Search result spec prec mz: 825.4314 Actual spec prec mz: 825.4314
Fragments matched per AA: 0.833 Proportion of top 20 peaks matched: 0.2
Intensity
1e+05
5e+04
0e+00
M/Z
500
1000
y12
y22
b6
y14
y15
b7
y16
y3_y1
b1
y3_
y5
y13
b13
y8
y9
b10_
y22_
y12
b12
B
Scatterplot of predicted elution time
Fitting R2: 0.878
Novel peptide residual Z score: −0.885
Number of peptides: 7864
Pred. hydrophobicity
50
25
0
Experimental scan number
0
25000
50000
75000
100000
C
Distributions of residuals from best−fit line
of predicted RT vs Expt. scan number
Line: Z score of novel peptide
Z: −0.885
count
2000
1500
1000
500
0
Z scores of residuals from best linear fit
−10
−5
0
5
10
